# Professor Taddei

**Published:** 1860-09

**Authors:** 


PROFESSOR TADDEI, well known as a chemist and cultivator of the natural sciences, died at Florence, on the twenty-ninth of May.

